# A Scoping Review of Respirator Literature and a Survey among Dental Professionals

**DOI:** 10.3390/ijerph17165968

**Published:** 2020-08-17

**Authors:** Marco Farronato, Elisa Boccalari, Ettore Del Rosso, Valentina Lanteri, Riaan Mulder, Cinzia Maspero

**Affiliations:** 1 Department of Biomedical, Surgical and Dental Sciences, School of Dentistry, University of Milan, 20122 Milan, Italy; marco.farronato@unimi.it (M.F.); valentina.lanteri@unimi.it (V.L.); 2 Fondazione IRCCS Ca’ Granda, Ospedale Maggiore Policlinico, via Francesco Sforza 35, 20122 Milan, Italy; 3 Department of Dentistry, Rho Hospital Stomatological Unit, Rho, 20100 Milano, Italy; eli.boccalari@gmail.com (E.B.); EDelrosso@asst-rhodense.it (E.D.R.); 4 Department of Restorative Dentistry, University of the Western Cape, Cape Town 80001, South Africa; rmulder@uwc.ac.za

**Keywords:** PPE, FFP2, dental professionals, headache, discomfort

## Abstract

Severe acute respiratory syndrome coronavirus 2 (SARS-CoV-2) virus was discovered in China in late 2019 and subsequently triggered a global pandemic. Dentists, like many other health professionals, are at an increased risk of contracting the virus as they work in close proximity to patients, especially when performing aerosol-generating procedures. Thus, in order for dentists to protect themselves and their patients, it is recommended that practitioners wear filtering facepiece 2 (FFP2) respirators. The prolonged use of these FFP2 respirators has been linked to several side effects. The aim of this paper is to assess the perceived experience associated with N95/FFP2 respirators based on the available literature and data collected through an online survey completed by Italian dental professionals. Articles were included up to May 2020 and literature searches were conducted through The National Library of Medicine, Cochrane Central Register of Controlled Trials, and Embase databases. The search terms included COVID-19, respirators, masks, and discomfort. An online survey was administered to 256 Italian dentists. The results from this survey were in agreement with the available literature. The findings concurred that the prolonged use of respirators was associated with headaches (47.5%), severe exertion and discomfort (50.8%), moderate concentration problems (54.3%), moderate breathing difficulties (63.5%), and consequently, an impaired work ability (85.5%). These findings were not influenced by the number of hours spent wearing the respirator. Despite several side effects, FFP2 respirators are fundamental in protecting dentists and their importance was acknowledged.

## 1. Introduction

The virus termed Severe acute respiratory syndrome coronavirus 2 (SARS-CoV-2) (COVID-19—Coronavirus disease 2019) was discovered in Wuhan, Hubei province, China late December 2019 [1]. It has triggered a global health crisis and in March 2020 [2], the World Health Organization (WHO) declared COVID-19 a pandemic. COVID-19 infections have exceeded 13 million, with at least 578,000 deaths reported [3]. The most common symptoms are fever, dry cough, fatigue, sometimes associated with sore throat, headaches, runny nose, loss of smell, and diarrhea. Scientists all over the world started studying COVID-19 and based on the data collected from previous similar infectious diseases, protocols were swiftly implemented in order to minimize disease transmission. This swift action included various protocols from the WHO [2] and Centers for Disease Control (CDC) [4] for healthcare workers, as they are at particular risk due to patient contact and potential asymptomatic individuals seeding the virus. 

Research on COVID-19 focused on the mode of transmission and disease progression in order to prevent contagion spread [5]. It was established that COVID-19 is spread by breathing and through direct or indirect contact with contaminated surfaces [6]. The fecal–oral route was also proposed by scientists because some studies identified viral RNA in patient feces; nevertheless, in the meanwhile, they became COVID-19 negative. However, up to the present day, no known cases of patients infected through fecal–oral route have been reported [7]. In vitro studies illustrated a potential transmission of the virus not only through droplets (small particles of moisture excreted by coughing or sneezing, >5 μm), but also through aerosol (smaller particles, <5 μm). The potential transmission of the virus is markedly increased with an increased duration of exposure as the environment becomes more concentrated with the virus [8]. Moreover, it was proved that COVID-19 could survive and remain suspended for up to three hours in aerosol, with a reduction in its infectious titer similar to the SARS-CoV-1 virus [6] (responsible for the SARS epidemic which took place in Asia from November 2002 to July 2003). One additional study from Lydia Bourouiba [9], published in the Journal of the American Medical Association (JAMA), question the proposed transmission model, by both WHO and CDC that based their theories on droplets. COVID-19 should be considered in aerosol form with an emphasis on the distance projection and survival of the viral particles. In fact, according to this study, it was suggested that the “turbulent puff cloud dynamic model” currently used could underestimate fundamental factors influencing the potential range and subsequent exposure to COVID-19. Bourouiba further suggest a new turbulent gas cloud dynamic model focused on moist and warm atmosphere that could trap COVID-19 and prevent droplets from evaporating for longer time periods. The study also proposed longer travel distances for droplets of up to 8 m, thus, increasing risks of COVID-19 spread [9]. Even though, at this stage, the validity of this concept requires further investigation with COVID-19, it is important to highlight the study to interrogate the engineering controls of the various health environments to assist in the protection for healthcare professionals.

Dental professionals, anesthetists, and ear-nose-and-throat specialists practice in very close proximity to patients with aerosol-generating procedures (AGPs). The term “aerosol generating procedures” (AGPs) encompass extubation, intubation, positive pressure ventilation (CPAP), tracheostomy, bronchoscopy, and upper gastrointestinal endoscopy. In the dental profession, ultrasonic devices, high-speed drilling, and 3-in-1 air-syringes are considered AGPs. Consequently, the use of a dental dam is highly recommended in order to reduce the spread of microbial loaded droplets [10]. These professions have an elevated risk to COVID-19, since AGPs facilitate the transmission of aerosol that contain saliva, blood, and other secretions. Dental professionals, in particular, due to their face-to–face contact and proximity for prolonged periods with patients, are at high risk of contamination through direct (as mentioned above) or indirect exposure to secretions, inhalation of AGPs, and mucosal contact with infected particles [11]. Moreover, as reported by Xu et al. [12], the high expression of ACE2 receptors in the oral mucosa make the oral cavity at potentially high risk for contraction of COVID-19. 

Along with four-handed dentistry with high volume air evacuation, hand hygiene, gowns, gloves, face shields, and goggles are fundamental in order to prevent COVID-19 from infecting dental professionals [13].

Thus, personal protective equipment (PPE) is a fundamental component in healthcare to reduce occupational exposure and risk to their health. A survey among Northern Italian dentists showed how the COVID-19 pandemic affected Italian dental practitioners and their use of PPE. The survey investigated the increasing or modifying use of PPE and their concerns regarding contracting COVID-19 during clinical activity [14]. 

The word “personal protective equipment” (PPE) encompasses all the protective equipment designed in order to safeguard workers from risks against health and safety. This includes gloves, mop caps, face shields, goggles, gowns, shoe covers, and the appropriate filtering face pieces. Many countries have advocated all of the aforementioned items during the performance of AGPs. COVID-19 is associated with a very small viral particle size and ability to infect the lower respiratory tract. 

A clear difference exists between examination earloop masks and surgical masks that are produced following the technical standards UNI EN 14638. Surgical masks are divided into three types in accordance with their filtering capacity (which is determined by the fabric, the bacterial filtering efficiency, and the facial seal) and resistance to splashes. Surgical masks under the standard UNI EN 14638 are divided into: Type I, II, and IIR (where R stands for resistance against spraying and humidity). For the technical standard ASTM F2100-11, surgical masks are divided into Levels 1 to 3 and are similar to the aforementioned UNI EN 14638. Type IIR/Level 3 surgical masks filter an average particle size of 2.7 µm and are resistant against droplets and splashes. Hand hygiene is always advised before donning, doffing, or adjusting any mask or respirator.

Filtering face pieces (FFP) or respirators, without an exhalation valve, provide two-way protection for the wearer and the patient. These FFP respirators provide a greater facial seal compared to surgical masks. FFP respirators are classified, according to European classification standard EN149:2001 based on their filtering efficiency of <0.3 μm droplets (FFP2 and FFP3). FFP2 can filter up to 94% of particles between 0.1 and 0.3 μm, extending this filtering capacity to 99% for particles of 0.75 μm and are made to the equivalent standard of the N95 respirator, which follows the NIOSH/FDA American standards. FFP3, meanwhile, filter up to 99% of particles with <0.3 μm of diameter and are similar to the American N99. The N99/FFP3 respirators have one-way valves.

However, there are conflicting approaches towards the efficacy and implementation of various levels of surgical masks versus N95/FFP2- and even N99/FFP3 respirators among different countries. The Italian “Istituto Superiore di Sanità” (ISS) produced a document [15] in which, foreseeing a future global lack of PPE, highlighted the necessity to rationalize their use. It is important to prioritize the most at risk healthcare workers, such as those working in AGPs and/or in a long-term exposure environment. The ISS document highlights the importance of adequate training that educates healthcare professionals of professional exposure risks. Such a training program would provide them with an overview of the suitable PPE for various clinical and infrastructure scenarios. N95/FFP2 and N99/FFP3 respirators with the other aforementioned PPE are recommended for AGPs. Surgical masks Type IIR/Level 3 are sufficient when giving direct assistance to COVID-19 patients, if filtering face pieces are not available.

Public Health England’s guidelines (Department of Health and Social Care in the United Kingdom) require an N99/FFP3 respirator in case of AGPs and in some procedures which carry a high risk of infection, such as patients’ intubation and extubation [16]. For direct care to COVID-19 patients, Type IIR/Level 3 surgical masks are recommended, and if there are none, an N95/FFP2 respirator is a suitable alternative [17,18,19,20].

Healthcare workers experienced masks and respirators differently based on their design features. The design features additionally allow their experience of discomfort and exertion to differ significantly during an 8 h work cycle [21]. The impact of prolonged respirator use, based on government regulations and PPE shortages, remains a growing problem for healthcare workers (HCW). In order to reduce the surface contamination of the respirator, an overlay with a medical face mask had been advised, purely to protect the respirator [22].

Moreover, hypercapnia-related problems after many hours of wearing these respirators have been a concern to healthcare workers (HCW) and have been studied under in vitro conditions [23] and in vivo studies for respiratory fit tests [24]. Laferty et al. focused on oxygen and carbon dioxide levels of subjects wearing N95 respirators in a test hood and results showed an increase in CO_2_ levels and a decrease in O_2_ levels due to the important breathing resistance offered by N95 respirators. Hypercapnia was also assessed, in another study, based on a normal working period of one hour [25]. During normal clinical activity, the healthcare workers experienced a decrease in O_2_ and an increase in CO_2_ levels with the two respirators assessed. Furthermore, several studies assessed the perceived effects of the respirators on the healthcare workers, but no studies have been completed with dental professionals.

The objectives of this paper were to evaluate the relevant findings in the literature regarding the rationale for use of an N95/FFP2 respirator related to AGPs particle sizes [26] that could contain the COVID-19 virus [6] in dental healthcare workers and assess the perceived symptoms experienced by dental professionals with the use of these respirators. The analysis of the survey results of the perceived symptoms from dental professionals would be valuable considering their elevated risk of transmission due to the various aerosol-generating procedures. This paper aims to compile the different survey questions based on a scoping review of the literature. All the literature was evaluated in the various search engines, although they could have evaluated responses from healthcare workers of different levels of surgical masks (Type I, II, and IIR) and disposable respirators (N95/FFP2). Additionally, a survey was completed with Italian dental professionals in order to evaluate their perceptions and perceived experience while wearing the advised N95/FFP2 respirators.

## 2. Materials and Methods 

Our review followed PRISMA guidelines for a scoping review. The studies included in this review matched the predefined criteria according to the PICOS (patients, intervention, comparator, outcomes, study design) process as reported in Table 1.

### 2.1. Eligibility Criteria

#### Inclusion Criteria

Types of studies and participants: cross sectional and case control studies focusing on healthcare workers.

Type of intervention: use of respirators and/or masks in daily practice and their clinical side effects such as discomfort, exertion, breathing difficulties, and headaches.

Language: Papers in English, Italian, and Spanish were included.

The exclusion criteria were reviews (historical or systematic), opinion letters, case reports, case series, congress abstracts, papers not written in English, Italian, and Spanish, and studies not reporting the positive or negative effects on the use of respirators and/or face masks.

### 2.2. Information Sources and Search

#### 2.2.1. Electronic Search

Three electronic databases were used in the search for studies satisfying the inclusion criteria for studies published until the 31 May 2020: The National Library of Medicine (MEDLINE via Pubmed), Cochrane Central Register of Controlled Trials, and Embase. The search was limited to human subjects. The search strategy employed was the following: ((COVID-19) AND (‘mask’ OR ‘respirator’ OR ‘PPE’, OR ‘protections’, OR ‘discomfort’, OR ‘headache’, OR ‘exertion’, OR ‘prevention’, OR ‘transmissions’, OR ‘dentist’)).

#### 2.2.2. Hand-Search

Cross-references were also considered together with the personal collections of the authors.

### 2.3. Study Selection

All the resulting articles were assessed for eligibility by EDR-MF reviewers independently, who screened the titles and abstracts for possible inclusion in the review, according to the inclusion criteria listed above. The agreement between the reviewers was also calculated (percentage agreement and kappa scores). Reviewers were first trained and calibrated for study screening against another reviewer (CM) with experience in conducting systematic reviews. Abstracts were excluded if they did not fulfil the inclusion criteria. To avoid the exclusion of potentially relevant articles, abstracts not providing the required information were included in the full-text analysis.

Full texts of potentially relevant studies were independently assessed by the same reviewers. Any disagreement was resolved by discussion between reviewers. Inter-observer agreement was assessed by means of kappa scores. Papers mentioning SARS-CoV-2 or COVID-19 transmission mode and PPE worn to protect from a possible contagion were included in this review, particularly the ones citing surgical masks and/or N95/FFP2 respirators. 

Duplicates were removed, articles were sorted by title, abstract, and then, full text. Once a paper was found eligible, its references were screened in order to find new papers for the revision.

Moreover, we submitted an anonymous online structured survey to 430 dental professionals belonging to an online community of dentists in order to assess their perceptions while wearing N95/FFP2 respirators. The assurance that the participants were members of the Italian Register of Dentists was given by the fact that it is a closed community and access is granted only if it is provided a registration to the professional order.

The 256 questionnaires obtained allow to estimate 47% headaches with an error at 6% and a 95% confidence level (CI95%).

The survey was structured in 3 main domains and we asked a total of 16 questions. The first part of the survey regarded demographics (age, gender), the second focused on their medical respiratory and smoking history and the number of hours spent wearing an N95/FFP2 respirator. The third part was about several possible perceived symptoms related to hypercapnia such as muscular pain, breathing difficulties, headaches, sleepiness, abnormally frequent urination, drop in attention levels, and consequently, drop in work ability. Questions about age, hypertension, headaches, muscular pain, and sleepiness were dichotomic (Yes/No questions), whereas questions about drop in attention levels, drop in work ability, breathing difficulties, and exertion were assigned a score ranging from 0 to 10 (0 = not experienced, 10 = extremely), categorized in three groups: 1–3 mild symptoms, 4–7 moderate symptoms, and 8–10 severe symptoms. All the questions referred to the experience of healthcare workers in the recent months of the pandemic situation wearing FFP2 respirators.

Inclusion criteria: all questionnaires returned, being at least 23 years old, and registered in the Italian National Register of Dentists.

### 2.4. Data Extraction

Data were extracted independently by two reviewers using specially designed data extraction forms. Contents of the data extraction included the following:

- Basic information of the trial: trial ID, title, authors, journal information;
- Eligibility reassessment: all the items in inclusion criteria, final decision;
- Study design: methods of randomization, allocation concealment, blinding, centers, country, funding;
- Participants’ information: inclusion and exclusion criteria of the trial, demographic characteristics (age, gender, etc.), number of participants in each group, baseline status;
- Intervention and comparison: details of the COVID-19 respirator and/or face masks use intervention and control groups, follow-up period, number of participants lost to follow-up and the reasons;
- Outcome: the name of outcome variables, assessment method, observation time and detailed results;
- Correspondence: contact address of the original authors.

Authors of the primary studies were consulted to obtain any further information not available in the paper. When the study results were published more than once or results were presented in multiple publications, the most complete dataset was identified, and data were included only once.

All data collected were analyzed using the software IBM SPSS Statistics 25 (Armonk, New York, NY, USA). Numbers and percentages of dichotomic variables were reported. Exact Binomial CI95% was reported for headache. Spearman’s rank correlation coefficient test (Spearman’s ρ) was calculated in order to assess whether there were correlations between variables. Chi square test was used to evaluate the relationship between sex and reported variables such as headache and concentration problems. All tests are to be considered with a 0.05 statistical significance level. 

## 3. Results

**Figure 1:**
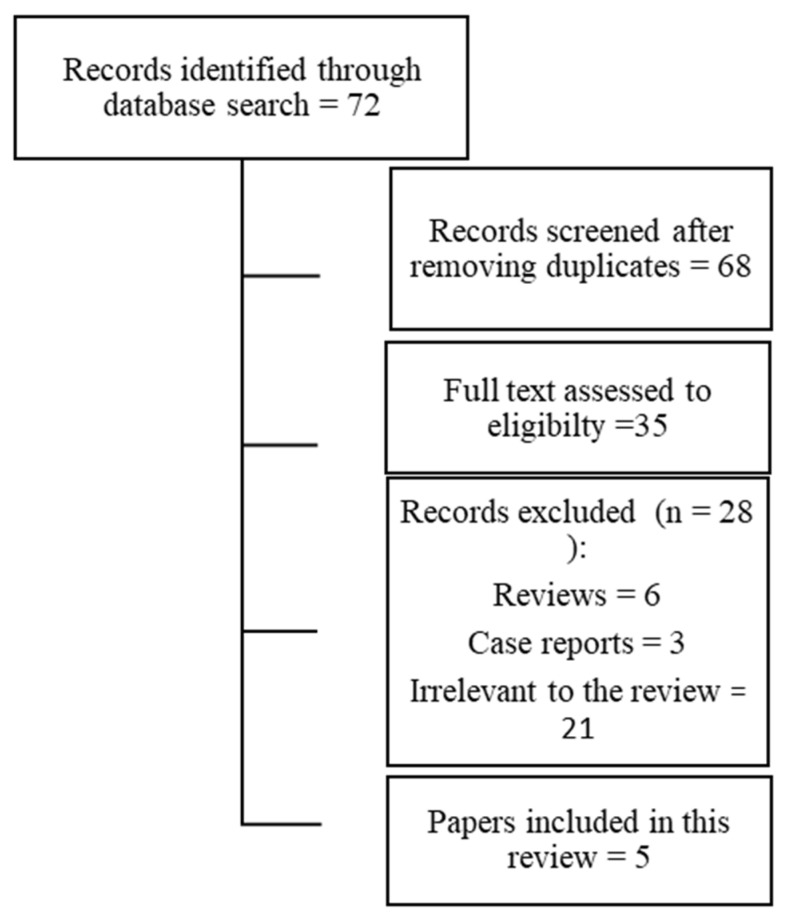
Flow diagram.

Following the search criteria in this scoping review, five papers were included, from three nations worldwide. Though the database research identified 72 documents, after having read the abstract and removal of the duplicates, only 35 papers were fully read. As shown by the flow diagram (Figure 1), 6 of them were reviews and therefore, were excluded and 24 did not comply with the criteria and were excluded. Results from the reviewed papers, following the outcomes, are shown in Table 2. All the available articles were about healthcare workers but there were not any dentists in the sample pool, since in the current literature, there were no studies among dentists experimenting the analyzed outcomes.

Two of these five studies [27,28] focused on headaches, particularly Lim et al. [27], who found 37% had headaches which presented after donning an N95/FFP2 respirator, and percentages up to 81% in Ong et al. [28]. Those high numbers were correlated to the numbers of daily hours wearing an FFP2/N95 respirator. 

Shenal et al. [21] investigated discomfort and exertion, through a self-reported scale and Borg exertion scale, in healthcare workers wearing masks and respirators for 8 h shifts with doffing periods every two hours. Their findings revealed a notable increase in discomfort and exertion over time even with doffing periods.

Rebmann et al. [29] studied the tolerance of operators regarding N95 respirators in a 12 h shift (with doffing periods) in a little sample of nurses working in a medical intensive care unit and investigated the reason behind each removal: in 22.1% of cases, nurses reported the removal was associated with discomfort and breathing difficulties.

The last article we analyzed, by Chughtai et al. [30], regarded medical masks, not respirators, worn by 148 doctors and nurses in three different hospitals in China and among the problems reported, there were breathing difficulties (16.9%), headaches (6.1%), and discomfort (9.5%) even just with surgical masks.

Table 3 represents the age distribution and each outcome of this survey for dental professionals based on the culmination of questions obtained from the scoping reviewed articles. The original sample size consisted of 430 dental professionals. Two-hundred-fifty-six (256) dental professionals (59.5%) fully completed the survey and their answers were indeed taken into consideration. The reasons for not answering three or more questions (thus, labeling the survey as incomplete) of the other colleagues were unknown to investigators (we suppose they were related to privacy or personal reasons). The final sample size with fully completed surveys was equally distributed between male (129) and female (127). All dental professionals wore the N95/FFP2 and 77.7% wore these N95/FFP2 respirators more than 4 h per day. Headaches were one of the most reported perceived side effects, reported by nearly half (47.5% CI95% 41.2–53.8) of the dental professional participants. Headaches were more reported in females than in males (55.9% of females vs 39.1% of males, *p* = 0.007). Headaches were negatively correlated with age (ρ = −0.151, *p* < 0.05) but not correlated with the number of hours spent wearing N95/FFP2 respirators. Moderate difficulties in breathing were reported by 162 participants (63.5% of the sample), with 20.8% of the participants experiencing severe breathing difficulties. Breathing difficulties were not correlated with the hours spent wearing N95/FFP2 respirators.

Moderate concentration problems are also reported by the survey with at least 139 participants (54.3%). Concentration problems became more severe for 66 dentists (25.8%) and medical histories indicated the presence of comorbidities (such as asthma, rhinitis, and OSAS) that has an effect on concentration problems among the participating dental professionals. 

Discomfort and exertion, which are common reports from the study, were a severe consequence of prolonged N95/FFP2 use for 50.8% of the sample (130 participants) and were related to gender, particularly with females (it was severe for 58.2% of females and 43.4% of males, *p* = 0.012) but not with the number of hours spent wearing respirators.

The presence of concentration problems, exertion, breathing difficulties, and headaches resulted in a moderate impaired working ability for 85.5% of our sample. In fact, an impaired working ability was strongly correlated to headaches (ρ = 0.212, *p* < 0.01), breathing difficulties (ρ = 0.566, *p* < 0.01), concentration problems (ρ = 0.748, *p* < 0.01), and exertion (ρ = 0.620, *p* < 0.01).

## 4. Discussion

This survey with Italian dental professionals and the literature concur that headaches appear to be the primary result of prolonged wear from respirators.

Lim et al. investigated a sample of 212 healthcare workers at healthcare facilities in China during the SARS outbreak in 2003–2004. Headaches were reported in 37.3% (79 HCW) from respirator use. Among these 79 HWC, 62.7% previously presented with no headaches and experienced it when they wore an N95/FFP2 respirator [27]. Moreover, they identified the prolonged duration of hours spent wearing an N95/FFP2 as an important risk factor for the development of headaches. These data are corroborated by another study conducted in Singapore during the COVID-19 pandemic, where in a sample of 158 HCW with comorbidities, 128 (81%) reported bilateral headaches. The majority of the respondents (81.3%) reported a headache within 60 min of donning an N95 respirator and resolved within 30 min for most (88.3%) participants [28]. The discomfort experienced by HCW, which developed into a headache, has been noted to have a bilateral localization matching the areas of contact from the face mask or goggles and their corresponding head straps. The authors suggested that pressure and traction from mask straps are likely to be concurrent in the pathogenesis of those headaches, along with hypercapnia, hypoxemia, and stress from the current pandemic situation and its consequent workload. The combination of N95 respirators and protective eyewear along with a prolonged use (>4 h/day) have been identified among the risk factors. Additionally, 82.8% of those with a de novo headache linked to PPE reported a slight decrease in work performance [28].

Data from our survey, indicating headaches as one of the main outcomes related to FFP2 wear, but not correlated to the hours spent wearing a respirator, is in contrast with previous reports from the literature [27,28]. Moreover, in our survey, there was a statistical difference in headaches among genders, whilst in Lim et al. [27], the difference was not significant.

Another significant side effect and relevant outcome in our survey was breathing difficulties, at least moderate for 63.5% of our sample, but not correlated with the hours spent wearing N95/FFP2 respirators. Chughtai et al. [30] detected only 12.2% breathing difficulties in their sample of 148 HCW. 

Such substantial differences among our studies are attributable to differences in the respirators worn by participants. Our sample is based on dentists wearing N95/FFP2 respirators without a valve, while the study by Chughtai et al. [30] was based on surgical disposable masks. Rebmann et al. [29], conversely, investigated the effects of N95 respirators but subjects reported removing respirators for a few minutes and in 22.1% of cases, the reason was discomfort linked to breathing difficulties. Rebmann et al. [29] was the only included article to measure O_2_ and CO_2_ levels, resulting in a statistical increase in CO_2_ levels compared with baseline measurements, even though this increase was not clinically relevant, and there were no changes in O_2_ levels. Unfortunately, the results were not assessed in correlation to the headaches, visual challenges, and lightheadedness investigated by the authors. 

Dental professionals usually take off masks between patients. We conjecture that the lack of correlation between headaches, breathing difficulties, and other symptoms with the numbers of hours spent wearing a respirator is the result of doffing periods between one patient and the subsequent one.

This also applies to Shenal et al. [21], who analyzed the level of discomfort and exertion in 27 HCW, noticing how even with predetermined doffing periods every 2 h, discomfort and exertion increased over time, still partially in contrast with our report, where this feature was not assessed despite them being commonly seen outcomes reported being severe from 50.8% of our sample. 

An impaired working ability (IWA), not assessed in the examined literature, was one of the strongest outcomes of our sample. It is interesting to note that IWA showed a strong positive correlation with breathing difficulties, concentration problems, and exertion. This could be explained with the perceived hypercapnia phenomena. Despite experiencing IWA and the other side effects, 49.2% of our group confirmed that FFP2 respirators are essential for the wellbeing of patients and healthcare workers, in agreement with Italian and international guidelines [15,19]. 

This survey is the first in Italy to assess a problem that, given the actual pandemic situation and consequent rise in the use of respirators, will be a common finding in future months. The results of our survey coincide with the current available literature. However, our study has some limitations, in fact, given the current pandemic situation, we were unable to test a control group of dentists wearing only facial masks. Moreover, it was a self-administered survey, not with an independent operator.

## 5. Conclusions

N95/FFP2 respirators without valves are currently recommended in order to prevent COVID-19 spread and preserve HCW wellbeing. N95/FFP2is acknowledged by the majority of dental professionals, although the majority experienced several perceived side effects. The practitioners wore the N95/FFP2 respirator for prolonged periods of time and the perceived side effects, such as breathing difficulties, headaches, and concentration problems, lead to an impaired working ability.

There are no well-structured studies that correlate the O_2_ and CO_2_ to the physical symptoms of healthcare workers. Thus, further studies are required with pulse oximetry to determine the level of hypercapnia in relation to headaches and an impaired working ability. Additionally, the pulse oximetry could assist in the assessment of valve respirators with a surgical mask in an attempt to decrease hypercapnia, since the literature is not conclusive.

## Figures and Tables

**Table 1:** PICOS—population, intervention, comparison, outcomes, study design.

Parameter	Inclusion Criteria	Exclusion Criteria
Population	Healthcare workers	Community people
Intervention	Use of respirators and/or masks in daily practice	
Comparison	Not applicable	Not applicable
Outcomes	Clinical side effects such as headache, discomfort, concentration problems, breathing difficulty, fatigue, and exertion	
Study design	Without restrictions	Papers not written in English, Italian, and Spanish; Reviews, opinion letters, case reports, case series, congress and abstracts; Studies not reporting the positive or negative effects of the use of masks and/or respirators.

**Table 2:** List of studies analyzed in the review.

Sample Size	Lim et al. [27]	Ong et al. [28]	Shenal et al. [21]	Rebmann et al. [29]	Chughtai et al. [30]
	*n* (%)	*n* (%)	*n* (%)	*n* (%)	*n* (%)
**Sample Size**	212	158	27	10	148
**Gender** **(M-male, F-female)**	M47 (22.2%) F165 (77.8%)	M47 (29.7%) F111 (70.3%)	M12 (44.4%) F15 (55.6%)	M1 (10%) F9 (90%)	27M (18.2%) 121F (81.8%)
**Age**	21–58 years (31 year mean age, SD = 7)	21–40 years: 138 (87.3%) >40 year: 20 (12.7%)	25–65 years (48 y mean age, SD = 11)	20–48 years	≤30 year: 41 (27.7%) 31–40 year: 68 (45.9%) ≥40 year: 39 (26.4%)
**Smoke**	Not assessed	2 (1.3%)	No (100%)	No (100%)	Not assessed
**Hypertension**	Not assessed	2 (1.3%)	Not assessed	Not assessed	Not assessed
**Asthma**	Not assessed	8 (5.1%)	Not assessed	Not assessed	Not assessed
**Respiratory Diseases**	Not assessed	Not assessed	Not assessed	Not assessed	No (100%)
**Daily Hours Wearing Masks or Respirators**	<4 h/day: 110 (51.9%) >4 h/day: 102 (48.1%)	1–4 h/day: 132 (83.5%) >4 h/day: 26 (16.5%)	8 h/day with doffing periods every 2 h	2 × 12 h	1–2 h/day: 1 (0.7%) 3–4 h/day: 8 (5.4%) 5–6 h/day: 40 (27%) 7–8 h/day: 80 (54.1%) >8 h/day: 19 (12.8%)
**Difficulty Breathing**	Not assessed	Not assessed	Not assessed	Assessed	18 (12.2%)
**Headache**	79 (37.3%)	128 (81%)	Not assessed	Assessed	9 (6.1%)
**Concentration Problems**	Not assessed	Not assessed	Not assessed	Not assessed	Not assessed
**Sleepiness**	Not assessed	Not assessed	Not assessed	Not assessed	Not assessed
**Exertion**	Not assessed	Not assessed	Assessed	Assessed	Not assessed
**Muscular Pain**	Not assessed	Noted but Not assessed	Not assessed	Not assessed	Not assessed
**Discomfort**	Not assessed	Noted but Not assessed	Assessed	Assessed	14 (9.5%)
**Pressure on Face**	Not assessed	Not assessed	Not assessed	Not assessed	25 (16.8%)

**Table 3:** Outcomes of the survey for dental professionals.

**Sample Size**	256					
**Gender**	M 129 (50.4%)	F 127 (49.6%)				
**Age**	23–29 years: 47 (18.5%)	30–39 years: 47 (18.5%)	40–49 years 72 (28.3%)	50–59 years 59 (23.2%)	>60 years 29 (11.4%)	Not Responding 2
**Smoke**	Yes 61 (23.8%)	No 169 (66%)	Ex smoker 26 (10.2%)			Not Responding 0
**Hypertension**	Yes 26 (10.2%)	No 230 (89.8%)				
**Respiratory Diseases**	No 199 (77.7%)	Asthma 12 (4.7%)	Allergic rhinitis 36 (14.1%)	Obstructive sleep apnea (OSA) 8 (3.1%)	Other answers 1 (0.4%)	Not Responding 0
**Daily Hours Wearing N95/FFP2 Respirators**	Less than 2 h/day: 9 (3.5%)	2–4 h/day: 48 (18.8%)	4–6 h/day 113 (44.1%)	6–8 h/day 86 (33.6%)		
**Difficulty Breathing**	Mild 40 (15.7%)	Moderate 162 (63.5%)	Severe 53 (20.8%)			Not Responding 1
**Headache**	Yes 121 (47.5%)	No 134 (52.5%)				Not Responding 1
**Concentration Problems**	Mild 51 (19.9%)	Moderate 139 (54.3%)	Severe 66 (25.8%)			Not Responding 0
**Sleepiness**	Yes 76 (29.8%)	No 179 (70.2%)				Not Responding 1
**Exertion**	Mild 26 (10.5%)	Moderate 99 (38.8%)	Severe 130 (50.8%)			Not Responding 0
**Muscular Pain**	Yes 78 (30.5%)	No 178 (69.5%)				Not Responding 0
**Increased Urination Stimulus**	Yes 44 (17.3%)	No 211 (82.7%)				Not Responding 1
**Impaired Working Ability**	Mild 37 (14.5%)	Moderate 139 (54.3%)	Severe 80 (31.2%)			Not Responding 0
**Are N95/FFP2 Respirators Essential for Protecting the Health of Patients and Your Own?**	Yes, FFP2 are essential 126 (49.2%)	No, there are better alternatives (FFP2 with valves) 56 (21.9%)	I don’t know 74 (28.9%)			Not Responding 0
**Pressure on Face**	Not assessed					

Legend: mild = 1<*>3, moderate = 4<*>7, severe= 8<*>10.
